# Inverse Fourier Transform in the Gamma Coordinate System

**DOI:** 10.1155/2011/285130

**Published:** 2010-10-26

**Authors:** Yuchuan Wei, Hengyong Yu, Ge Wang

**Affiliations:** ^1^Department of Radiation Oncology, Wake Forest University School of Medicine, Winston-Salem, NC 27157, USA; ^2^Biomedical Imaging Division, VT-WFU School of Biomedical Engineering and Sciences, Wake Forest University Health Sciences, Winston-Salem, NC 27157, USA; ^3^Department of Radiology, Division of Radiologic Sciences, Wake Forest University Health Sciences, Winston-Salem, NC 27157, USA; ^4^Biomedical Imaging Division, VT-WFU School of Biomedical Engineering and Sciences, Virginia Tech., Blacksburg, VA 24061, USA

## Abstract

This paper provides auxiliary results for our general scheme of computed tomography. In 3D parallel-beam geometry, we first demonstrate that the inverse Fourier transform in different coordinate systems leads to different reconstruction formulas and explain why the Radon formula cannot directly work with truncated projection data. Also, we introduce a gamma coordinate system, analyze its properties, compute the Jacobian of the coordinate transform, and define weight functions for the inverse Fourier transform assuming a simple scanning model. Then, we generate Orlov's theorem and a weighted Radon formula from the inverse Fourier transform in the new system. Furthermore, we present the motion equation of the frequency plane and the conditions for sharp points of the instantaneous rotation axis. Our analysis on the motion of the frequency plane is related to the Frenet-Serret theorem in the differential geometry.

## 1. Introduction

In [1], we developed a general scheme for 2D and 3D, parallel- and divergent-beam computed tomography (CT). Different from traditional Radon's or Tuy's formulas starting with the inverse Fourier transform in the spherical coordinate system, our new framework is based on an instantaneous cylindrical coordinate system. However, in [1], the coordinate system was not formally defined, and the Jacobian of the transform was not explicitly calculated. Given the basic role of our general scheme in CT theory and some confusion in the literature [2–5], here, we treat the Γ coordinate system in a mathematically strict way. 

The organization of this paper is as follows. In Section 2, we demonstrate how the inverse Fourier transform formula generates different reconstruction formulas, with a special attention on different properties of projection truncation. In Section 3, we define the Γ coordinate system and deduce its Jacobian factor. In Section 4, we study the motion of the frequency plane and present the condition for sharp points of the instantaneous rotation axis. In Section 5, a simplified model for helical CT reconstruction is given. In the appendixes, we present a weighted Λ reconstruction formula, a weighted Radon's formula, a deduction of Orlov's formula, and an alternative way to study the motion of the frequency plane. For convenience, we use the same notations as in [1]. 

## 2. Inverse Fourier Transform in Commonly Used Coordinate Systems

In this section, we write the inverse Fourier transform in commonly used coordinate systems, derive corresponding reconstruction formulas, and demonstrate their difference on truncation of projection data. 

**Figure 1:**
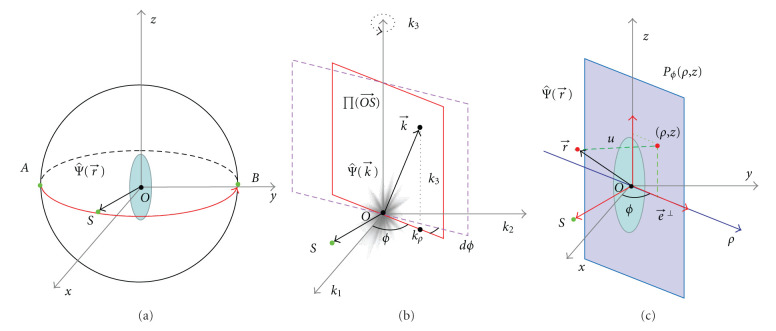
3D reconstruction problem in parallel-beam geometry and the cylindrical coordinate system. (a) A parallel-beam scans an object along a semicircle locus on the unit sphere; (b) In the frequency domain, when *ϕ*_0_ increases from 0 to *π*, the frequency plane *ϕ* = *ϕ*_0_ scans every point exactly once except for the points on the *k*_3_ axis, and the end point of the normal vector $\vec{OS}$ moves along the equator from (0, −1,0) to (0,1,0); (c) For simplicity, we suppose that the detector panel passes through the origin.

As shown in Figure 1, in the 3D space $R^{3},\;\Psi (\vec{r})$ is an object function to be reconstructed, whose Fourier transform is $\hat{\Psi }(\vec{k}).$ According to Fourier analysis, we have

(1) $$\begin{array}{cc} & \Psi \left(\vec{r}\right)=\overset{\infty }{\underset{-\infty }{\int }}\overset{\infty }{\underset{-\infty }{\int }}\overset{\infty }{\underset{-\infty }{\int }}\hat{\Psi }\left(\vec{k}\right)\exp\;\left(2\pi i\vec{k}\cdot \vec{r}\right)dk_{1}dk_{2}dk_{3}, \end{array}$$

where $\vec{r}=(x,\;y,\;z)$ and $\vec{k}=(k_{1},k_{2},k_{3})$ are 3D vectors in the real space and Fourier domain, respectively. The unit vectors along the three axes are $\vec{\text{i}},\vec{\text{j}},$ and $\vec{\text{k}}$. Note that the frequency vector $\vec{k}$ and the unit vector $\vec{\text{k}}$ are different. This formula states that to reconstruct an 3D object $\Psi (\vec{r}),$ we need its Fourier transform $\hat{\Psi }(\vec{k})$ in the whole 3D space. In X-ray CT, the Fourier transform $\hat{\Psi }(\vec{k})$ can be calculated when sufficiently many parallel projections are measured. 

Now, let us specify the simplest 3D reconstruction problem. In Figure 1(a), *S*(*ϕ*) = *S*(sin  *ϕ*, −cos  *ϕ*, 0) with *ϕ* ∈ [0, *π*] is a point on a great semicircle of the unit sphere. In the 3D space, we define the unit vectors $\vec{e}=\vec{OS}=(\sin\;\;\phi ,-\cos\;\;\phi ,0)$ and ${\vec{e}}^{⊥}=(\cos\;\;\phi ,\sin\;\;\phi ,0).$ Then, $\vec{e},\;{\vec{e}}^{⊥}$ and $\vec{\text{k}}$ are another set of orthogonal unit vectors. As shown in Figure 1(c), the new components of the vector $\vec{r}$ can be denoted as

(2) $$\begin{array}{cc} & u=\vec{r}\cdot \vec{e},\;\;\rho =\vec{r}\cdot {\vec{e}}^{⊥},\;\;z=\vec{r}\cdot \vec{\text{k}}. \end{array}$$

The projection at point *S* is defined by

(3) $$\begin{array}{cc} & P_{S}\left(\rho ,z\right)=P_{\phi }\left(\rho ,z\right)=\overset{\infty }{\underset{-\infty }{\int }}\Psi \left(u\vec{e}+\rho {\vec{e}}^{⊥}+z\vec{\text{k}}\right)du. \end{array}$$

Our purpose is to reconstruct $\Psi (\vec{r})$ when *P*_*ϕ*_(*ρ*, *z*) is known for all *ϕ* ∈ [0, *π*]. Let us see how the selection of a coordinate system will determine the reconstruction formula, paying an attention to longitudinal data truncation. 

### 2.1. Signed Cylindrical Coordinate System

In a cylindrical coordinate system, the inverse Fourier transform can be rewritten as

(4) $$\begin{array}{cc} & \Psi \left(\vec{r}\right)=\overset{\pi }{\underset{0}{\int }}\overset{\infty }{\underset{-\infty }{\int }}\overset{\infty }{\underset{-\infty }{\int }}\hat{\Psi }\left(\vec{k}\right)\exp\;\left(2\pi i\vec{k}\cdot \vec{r}\right)\left|k_{\rho }\right|dk_{\rho }dk_{3}d\phi , \end{array}$$

where (*k*_*ρ*_, *k*_3_, *ϕ*) with −*∞* < *k*_*ρ*_ < *∞*, −*∞* < *k*_3_ < *∞*, 0 ≤ *ϕ* < *π* is the signed cylindrical coordinates of $\vec{k}$. As shown in Figure 1(b), we have

(5) $$\begin{array}{cc} & \vec{k}=\vec{k}\left(k_{\rho },k_{3},\phi \right)=k_{\rho }{\vec{e}}^{⊥}\left(\phi \right)+k_{3}\vec{\text{k}}. \end{array}$$

When *ϕ*_0_ increases from 0 to *π*, the plane *ϕ* = *ϕ*_0_ passes through every frequency point exactly once (except for the points on the axis *Ok*_3_ which have a zero measure), and the normal vector $\vec{OS}$ of the plane *ϕ* = *ϕ*_0_ moves along a great semicircle on the unit sphere with the starting point A(0, −1,0) and the end point B(0,1, 0). Note that the coordinate transform from (*k*_1_, *k*_2_, *k*_3_) to (*k*_*ρ*_, *k*_3_, *ϕ*) is one to one (except for the points on the axis *Ok*_3_ with zero measure), and the absolute value of Jacobian is |*k*_*ρ*_|. 

 From the parallel projection *P*_*ϕ*_(*ρ*, *z*), we can calculate the Fourier transform of the object

(6) $$\begin{array}{cc} \hat{\Psi }\left(k_{\rho },k_{3},\phi \right) & ={\hat{P}}_{\phi }\left(k_{\rho },k_{3}\right)\\ & =\overset{\infty }{\underset{-\infty }{\int }}\overset{\infty }{\underset{-\infty }{\int }}P_{\phi }\left(\rho ,z\right)\exp\;\left[-2\pi i\left(k_{\rho }\rho +k_{3}z\right)\right]d\rho dz. \end{array}$$

Substituting (6) into (4), we have

(7) $$\begin{array}{cc} & \Psi \left(\vec{r}\right)=\overset{\pi }{\underset{0}{\int }}\overset{\infty }{\underset{-\infty }{\int }}\overset{\infty }{\underset{-\infty }{\int }}{\hat{P}}_{\phi }\left(k_{\rho },k_{3}\right)\exp\;\left(2\pi i\vec{k}\cdot \vec{r}\right)\left|k_{\rho }\right|dk_{\rho }dk_{3}d\phi . \end{array}$$

The reconstruction scheme behind this formula can be denoted as

(8) $$\begin{array}{cc} & P_{\phi }\left(\rho ,z\right)\to \hat{\Psi }\left(\vec{k}\right)\to \Psi \left(\vec{r}\right), \end{array}$$

which indicates that we first calculate all the Fourier components from projections and then reconstruct the object using the inverse Fourier transform. In this process, the object is reconstructed as a whole body. 

In fact, Formula (7) can be written in the filtered backprojection form as

(9) $$\begin{array}{cc} & \Psi \left(\vec{r}\right)=\overset{\pi }{\underset{0}{\int }}{\tilde{P}}_{\phi }\left(\rho ,z\right)d\phi , \end{array}$$

where the filtered projection is given by

(10) $$\begin{array}{cc} & {\tilde{P}}_{\phi }\left(\rho ,z\right)\\ & \;=\overset{\infty }{\underset{-\infty }{\int }}\overset{\infty }{\underset{-\infty }{\int }}{\hat{P}}_{\phi }\left(k_{\rho },k_{3}\right)\exp\;\left(2\pi i\vec{k}\cdot \vec{r}\right)\left|k_{\rho }\right|dk_{\rho }dk_{3}\\ & \;=\overset{\infty }{\underset{-\infty }{\int }}\overset{\infty }{\underset{\infty }{\int }}{\hat{P}}_{\phi }\left(k_{\rho },k_{3}\right)\exp\;\left(2\pi ik_{\rho }\rho \right)\exp\;\left(2\pi ik_{3}z\right)\left|k_{\rho }\right|dk_{\rho }dk_{3}\\ & \;=P_{\phi }\left(\rho ,z\right)*\frac{1}{-2\pi^{2}\rho^{2}}. \end{array}$$

The symbol ∗ denotes the 1D convolution operation about variable *ρ*. Since the filtration is along the horizontal direction only, the projection can be longitudinally truncated, that is, a plane with a specified *z* value can be reconstructed independently. The reconstruction scheme behind (9) with (10) can be denoted by

(11) $$\begin{array}{cc} & \hat{\Psi }\left(\vec{k}\right)\to P_{\phi }\left(\rho ,z\right)\to \Psi \left(\vec{r}\right), \end{array}$$

which states that certain frequency components form the projection, and the object can be reconstructed directly from projections. 

 When projections are longitudinally truncated, the Fourier transform cannot be calculated. How and why can we reconstruct a part of the object? 

 The answer is as follows. What we want to reconstruct is the object $\Psi (\vec{r})$ instead of the Fourier transform $\hat{\Psi }(\vec{k})$. Though we cannot calculate every single frequency component, we can calculate the contribution of the frequency components on the whole frequency plane (as well as its neighborhood) to a point to be reconstructed, see (10). In the aforementioned case, projections contain as all frequency components as the object does, but the ratio of the frequency components is not suitable. Then, the only thing we need to do is to perform a 1D filtration to adjust this ratio. An analog is that the Fourier components can be considered as raw material, projections a semifinished product, and the reconstructed object the final product. If we use the semifinished product to make a product, we do not need change it back to the raw material. The role of Fourier analysis is to tell us how far from the semifinished product to the final product. Simply speaking, *the essence of image reconstruction from projections is filtration.* Therefore, a suitable coordinate system should show the character of the filtration in an easy way.

### 2.2. Signed Spherical Coordinate System

**Figure 2:**
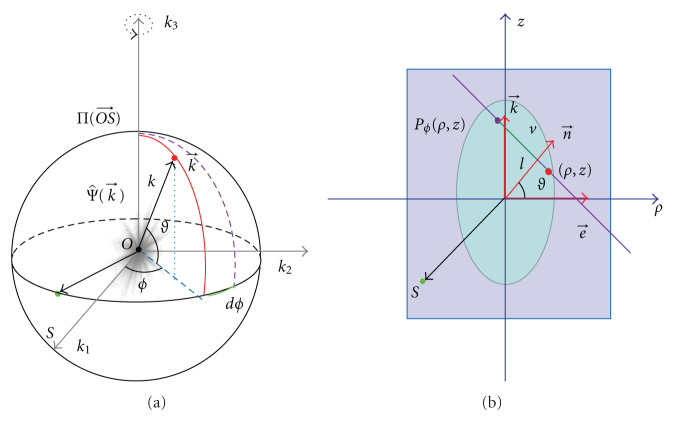
The sphere coordinate system and the detector plane. (a) The sphere coordinate system in the Fourier domain, and (b) the relationship between projections and the Radon transform.

In the signed spherical coordinate system shown in Figure 2, the inverse Fourier transform can be rewritten as

(12) $$\begin{array}{cc} & \Psi \left(\vec{r}\right)=\overset{\pi }{\underset{0}{\int }}\overset{\pi /2}{\underset{-\pi /2}{\int }}\overset{\infty }{\underset{-\infty }{\int }}\hat{\Psi }\left(\vec{k}\right)\exp\;\left(2\pi i\vec{k}\cdot \vec{r}\right)k^{2}\cos\;\vartheta dkd\vartheta d\phi , \end{array}$$

where (*k*, *ϑ*, *ϕ*) with −*∞* < *k* < *∞*, −*π*/2 < *ϑ* < *π*/2, 0 ≤ *ϕ* < *π* is the signed spherical coordinates of $\vec{k}$. 

For convenience, we denote $\vec{k}=\vec{k}(k,\vartheta ,\phi )=k\vec{n}$, where $\vec{n}=\vec{n}(\vartheta ,\phi )={\vec{e}}^{⊥}(\phi )\cos\;\vartheta +\vec{\text{k}}\sin\;\vartheta$ is a unit vector. 

 For a unit vector $\vec{n}=\vec{n}(\vartheta ,\phi )$, and a real number *l* ∈ (−*∞*, *∞*), the Radon transform of the object is defined as an integral

(13) $$\begin{array}{cc} & R\Psi \left(l,\vec{n}\right)=R\Psi \left(l,\vartheta ,\phi \right)=\iint_{\vec{r}\cdot \vec{n}=l}\Psi \left(\vec{r}\right)d^{2}\vec{r}, \end{array}$$

on the plane described by $\vec{r}\cdot \vec{n}=l$. 

The Fourier transform can be calculated from the Radon transform by

(14) $$\begin{array}{cc} & \hat{\Psi }\left(\vec{k}\right)=\hat{\Psi }\left(k\vec{n}\right)=\overset{\infty }{\underset{-\infty }{\int }}R\Psi \left(l,\vec{n}\right)\exp\;\left(-2\pi ikl\right)dl. \end{array}$$

Thus, the inverse Fourier transform (12) becomes Radon's formula

(15) $$\begin{array}{cc} \Psi \left(\vec{r}\right) & =-\frac{1}{4\pi^{2}}\overset{\pi }{\underset{0}{\int }}\overset{\pi /2}{\underset{-\pi /2}{\int }}{\;\frac{\partial^{2}}{\partial l^{2}}|}_{l=\vec{r}\cdot \vec{n}}R\Psi \left(l,\vec{n}\right)\cos\;\vartheta d\vartheta d\phi \\ & =-\frac{1}{4\pi^{2}}\nabla^{2}\overset{\pi }{\underset{0}{\int }}\overset{\pi /2}{\underset{-\pi /2}{\int }}{\;R\Psi \left(l,\vec{n}\right)|}_{l=\vec{r}\cdot \vec{n}}\cos\;\vartheta d\vartheta d\phi \\ & =-\frac{1}{8\pi^{2}}\nabla^{2}\overset{}{\underset{\Omega }{\int }}{\;R\Psi \left(l,\vec{n}\right)|}_{l=\vec{r}\cdot \vec{n}}\;d\vec{n}, \end{array}$$

where ∇^2^ is the Laplace operator, and Ω is the unit sphere. 

Radon's formula tells us that to reconstruct the object at a point, what we need is the Radon transform of the planes through the point and its neighborhood. However, what we measure by the detector is line integrals along X-rays instead of the Radon transform [6] but it can be calculated from projections by

(16) $$\begin{array}{cc} R\Psi \left(l,\vec{n}\right) & =R\Psi \left(l,\vartheta ,\phi \right)\\ & =\overset{\infty }{\underset{-\infty }{\int }}P_{\phi }\left(l\cos\;\vartheta -v\sin\;\vartheta ,l\sin\;\vartheta +v\cos\;\vartheta \right)dv. \end{array}$$

We call (16) the first relation between the Radon transform and parallel projection. 

Formulas (15) and (16) form a scheme to reconstruct the object from its parallel projections. Here, the projection is not allowed to be longitudinally truncated. From Section 2.1, however, we have seen that the object can be reconstructed slice by slice from parallel projections. Why is the truncation not allowed when using the Radon formula? 

For comparison, we rewrite Radon's formula in the filtered backprojection format

(17) $$\begin{array}{cc} & \Psi \left(\vec{r}\right)=\overset{\pi }{\underset{0}{\int }}{\tilde{P}}_{\phi }\left(\rho ,z\right)d\phi . \end{array}$$

with

(18) $$\begin{array}{cc} {\tilde{P}}_{\phi }\left(\rho ,z\right) & =\overset{\infty }{\underset{-\infty }{\int }}\overset{\pi /2}{\underset{-\pi /2}{\int }}\hat{\Psi }\left(\vec{k}\right)\exp\;\left(2\pi i\vec{k}\cdot \vec{r}\right)k^{2}\cos\;\vartheta dkd\vartheta \\ & =\frac{1}{-4\pi^{2}}\overset{\pi /2}{\underset{-\pi /2}{\int }}\;{\;\frac{\partial^{2}}{\partial l^{2}}|}_{\begin{array}{c} l=\vec{r}\cdot \vec{n}\\ =\rho \cos\;\vartheta +z\sin\;\vartheta \end{array}}R\Psi \left(l,\vec{n}\right)\cos\;\vartheta d\vartheta . \end{array}$$

Comparing (18) with (10), we have the following relation between the Radon transform and parallel-beam projections

(19) $$\begin{array}{cc} & \frac{1}{-4\pi^{2}}\overset{\pi /2}{\underset{-\pi /2}{\int }}{\;\frac{\partial^{2}}{\partial l^{2}}|}_{\begin{array}{c} l=\vec{r}\cdot \vec{n}\\ =\rho \cos\;\vartheta +z\sin\;\vartheta \end{array}}R\Psi \left(l,\vec{n}\left(\vartheta ,\phi \right)\right)\cos\;\vartheta d\vartheta \\ & \;\;=P_{\phi }\left(\rho ,z\right)*\frac{1}{-2\pi^{2}\rho^{2}}. \end{array}$$

We call this the second relation between the Radon transform and parallel-beam projections. Based on the relationship between fan-beam and parallel-beam projections [1], the right hand side of (19) can be expressed in terms of cone-beam projections as well. 

Substituting (16) into (19), we get an identity of parallel projections

(20) $$\begin{array}{cc} & \frac{1}{-4\pi^{2}}\overset{\pi /2}{\underset{-\pi /2}{\int }}{\;\frac{\partial^{2}}{\partial l^{2}}|}_{l=\rho \cos\;\vartheta +z\sin\;\vartheta }\\ & \;\;\times \overset{\infty }{\underset{-\infty }{\int }}P_{\phi }\left(l\cos\;\vartheta -v\sin\;\vartheta ,l\sin\;\vartheta +v\cos\;\vartheta \right)dv\cos\;\vartheta d\vartheta \\ & \;=P_{\phi }\left(\rho ,z\right){}^{*}\frac{1}{-2\pi^{2}\rho^{2}}. \end{array}$$

Now, we see that reconstruction of an object from parallel-beam projections does allow truncation of projection data but to verify the identity (20) needs the information on the whole projection domain. Reconstruction from parallel-beam projections using the Radon formula means numerically verifying this identity. We point out that if the projection *P*_*ϕ*_(*ρ*, *z*) is replaced by a general function with two variables, this identity holds true as well; see Appendix A. 

Similarly, in cone-beam CT, reconstruction from cone-beam projections using the Radon formula, that is, the Grangeat's framework, means numerically verifying the cone-beam version of this identity. This is the reason why the Grangeat framework cannot deal with the long object problem. 

We see that the Radon formula comes from the inverse Fourier transform in the signed spherical coordinate system. In this simplest reconstruction problem, there are two shortcomings with the Radon formula: (1) what we measure with X-ray is line integrals rather than planar integrals, (2) the projection cannot be longitudinally truncated.

### 2.3. Standard Spherical Coordinate System

In a standard spherical coordinate system, the inverse Fourier transform is written as

(21) $$\begin{array}{cc} & \Psi \left(\vec{r}\right)=\overset{2\pi }{\underset{0}{\int }}\overset{\pi /2}{\underset{-\pi /2}{\int }}\overset{\infty }{\underset{0}{\int }}\hat{\Psi }\left(\vec{k}\right)\exp\;\left(2\pi i\vec{k}\cdot \vec{r}\right)k^{2}\cos\;\vartheta dkd\vartheta d\phi , \end{array}$$

where (*k*, *ϑ*, *ϕ*) with  0 ≤ *k* < *∞*, −*π*/2 < *ϑ* < *π*/2, 0 ≤ *ϕ* < 2*π* is the standard spherical coordinates of $\vec{k}$, as shown in Figure 2. 

 From the well-known generalized function relations [7]

(22) $$\begin{array}{c} \delta \left(l\right)=\overset{\infty }{\underset{-\infty }{\int }}\exp\;\left(2\pi ikl\right)dk,\\ \frac{i}{\pi l}=\overset{\infty }{\underset{-\infty }{\int }}\mathrm{sgn}\;\left(k\right)\exp\;\left(2\pi ikl\right)dk, \end{array}$$

we have

(23) $$\begin{array}{c} \frac{1}{2}\delta \left(l\right)+\frac{i}{2\pi l}=\overset{\infty }{\underset{0}{\int }}\exp\;\left(2\pi ikl\right)dk,\\ \frac{1}{2}\delta^{\prime \prime }\left(l\right)+\frac{i}{\pi l^{3}}=-4\pi^{2}\overset{\infty }{\underset{0}{\int }}k^{2}\exp\;\left(2\pi ikl\right)dk. \end{array}$$

Based on the convolution theorem, we have

(24) $$\begin{array}{cc} & \overset{\infty }{\underset{0}{\int }}\hat{\Psi }\left(\vec{k}\right)\exp\;\left(2\pi i\vec{k}\cdot \vec{r}\right)k^{2}dk\\ & \;\;=\overset{\infty }{\underset{0}{\int }}\hat{\Psi }\left(k\vec{n}\right)\exp\;\left(2\pi ikl\right)k^{2}dk\\ & \;\;=-\frac{1}{8\pi^{2}}\frac{\partial^{2}}{\partial^{2}l}R\Psi \left(l,\vec{n}\right)-\frac{i}{4\pi^{3}l^{3}}*R\Psi \left(l,\vec{n}\right). \end{array}$$

Therefore, the inverse Fourier transform becomes

(25) $$\begin{array}{cc} \Psi \left(\vec{r}\right) & =-\frac{1}{8\pi^{2}}\overset{2\pi }{\underset{0}{\int }}\overset{\pi /2}{\underset{-\pi /2}{\int }}{\;\frac{\partial^{2}}{\partial l^{2}}R\Psi \left(l,\vec{n}\right)|}_{l=\vec{r}\cdot \vec{n}}\cos\;\vartheta d\vartheta d\phi \\ & \;-\frac{i}{4\pi^{3}}\overset{2\pi }{\underset{0}{\int }}\overset{\pi /2}{\underset{-\pi /2}{\int }}{\;\left[R\Psi \left(l,\vec{n}\right)*\frac{1}{l^{3}}\right]|}_{l=\vec{r}\cdot \vec{n}}\cos\;\vartheta d\vartheta d\phi . \end{array}$$

Based on the odd and even symmetry, we have the Radon formula again [6]

(26) $$\begin{array}{cc} & \Psi \left(\vec{r}\right)=-\frac{1}{4\pi^{2}}\overset{\pi }{\underset{0}{\int }}\overset{\pi /2}{\underset{-\pi /2}{\int }}{\;\frac{\partial^{2}}{\partial l^{2}}R\Psi \left(l,\vec{n}\right)|}_{l=\vec{r}\cdot \vec{n}}\cos\;\theta d\theta d\phi . \end{array}$$

The difference between the signed and standard spherical coordinate systems is little, and the signed spherical coordinate system leads to Radon's formula more easily. 

It is important to underline that the inverse Fourier transform may yield various reconstruct formulas in different coordinate systems and allow different degrees of data truncation. Among the commonly used coordinate systems, the signed cylindrical coordinate system is the most convenient one to solve the simplest 3D reconstruction problem. To handle the general 3D reconstruction problem, in the following we will introduce a variant of the signed cylindrical coordinate system, which is referred to as the Γ coordinate system.

## 3. Γ Coordinate System and Its Jacobian Factor

What plays an important role in the 3D reconstruction field is a variant of the cylindrical coordinate system, which can be named the Γ coordinate system. 

**Figure 3:**
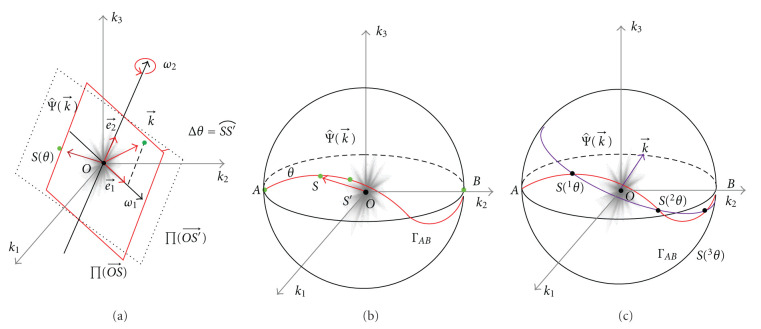
Nonuniqueness of the representation of a point in the Γ coordinates. (a) The Γ coordinate system, (b) a general curve Γ_AB_ on the unit sphere, and (c) the intersections of Γ_AB_ and the great circle orthogonal to a given vector $\vec{k}.$ When *S* moves from A to B Γ_AB_, some region may be scanned more than once.

As shown in Figure 3(b),  Γ_AB_ is a three-times differentiable curve connecting points A(0,−1,0) and B(0,1,0) on the unit sphere, whose length is *θ*_0_ ≥ *π*.  *S*(*θ*) is a point on Γ_AB_ parameterized by *θ* ∈ [0, *θ*_0_] the length of the segment AS. Let us introduce three orthogonal unit vectors

(27) $$\begin{array}{c} {\vec{e}}_{3}=\vec{OS},\\ {\vec{e}}_{1}=\frac{d}{d\theta }{\vec{e}}_{3},\\ {\vec{e}}_{2}={\vec{e}}_{3}\times {\vec{e}}_{1}, \end{array}$$

where ${\vec{e}}_{1}$ represents the tangential direction, ${\vec{e}}_{2}$ the instantaneous rotation axis of the frequency plane. Note that they are functions of $\theta =\int_{A}^{S}\left|d{\vec{e}}_{3}\right|$ (see Figures 3(a) and 3(b)).

In the Fourier domain, we call the plane through the origin *O* and orthogonal to the vector $\vec{OS}$ the frequency plane, denoted as $\prod \left(\vec{OS}\right)$. Every point $\vec{k}\in \prod \left(\vec{OS}\right)$ can be expressed as

(28) $$\begin{array}{cc} & \vec{k}=\omega_{1}{\vec{e}}_{1}+\omega_{2}{\vec{e}}_{2}, \end{array}$$

with $\omega_{1}=\vec{k}\cdot {\vec{e}}_{1},\;\;\omega_{2}=\vec{k}\cdot {\vec{e}}_{2}.$

More generally, for every given triplet (*ω*_1_, *ω*_2_, *θ*) with *ω*_1_ ∈ (−*∞*, *∞*),  *ω*_2_ ∈ (−*∞*, *∞*),  *θ* ∈ [0, *θ*_0_], we can define a 3D vector

(29) $$\begin{array}{cc} & \vec{k}=\vec{k}\left(\omega_{1},\omega_{2},\theta \right)=\omega_{1}{\vec{e}}_{1}\left(\theta \right)+\omega_{2}{\vec{e}}_{2}\left(\theta \right), \end{array}$$

and the triplet (*ω*_1_, *ω*_2_, *θ*) is a set of Γ coordinates of the point $\vec{k}.$

However, a given point $\vec{k}\in R^{3}$ may be represented by several sets of Γ coordinates, because when *S*(*θ*) moves along Γ_AB_, the rotation axis ${\vec{e}}_{2}$ keeps changing, and hence the frequency plane $\prod \left(\vec{OS}\right)$ may scan the given point $\vec{k}$ more than once. The number of times of the point being scanned, denoted as $J(\vec{k}),$ equals the number of intersections between the curve Γ_AB_ and the great circle orthogonal to the vector $\vec{k}$ (Figure 3(c)). To find the all intersections, one can solve the equation

(30) $$\begin{array}{cc} & \vec{k}\cdot {\vec{e}}_{3}\left(\theta \right)=0, \end{array}$$

to obtain the solutions θ1,  θ2,  …,θJ(k⃗). For every solution, such as ^1^*θ*, one can further have ω11=k⃗·e⃗1(θ1),  ω21=k⃗·e⃗2(θ1). Thus, one gets the $J(\vec{k})$ groups of coordinates

(31) k⃗=k⃗(ω11,ω21,θ1)=k⃗(ω12,ω22,θ2)=⋯=k⃗(ω1J(k⃗),ω2J(k⃗),θJ(k⃗)).

We call this new system the Γ coordinate system, since the system is based on the curve Γ_AB_. This coordinate system is a variant of the cylindrical system. For a differential arc *SS*′, the new system is similar to a cylindrical coordinate system if the scanned volume is considered. When Γ_*AB*_ is a great semicircle, the Γ coordinate system becomes the cylindrical coordinate system. 

In the Γ coordinate system, the inverse Fourier transform can be expressed as

(32) $$\begin{array}{cc} \Psi \left(\vec{r}\right) & =\overset{\theta_{0}}{\underset{0}{\int }}\overset{\infty }{\underset{-\infty }{\int }}\overset{\infty }{\underset{-\infty }{\int }}W\left(\omega_{1},\omega_{2},\theta \right)\exp\;\;\left(2\pi i\vec{k}\cdot \vec{r}\right)\\ & \;\;\;\;\;\;\times \hat{\Psi }\left(\vec{k}\right)\left|\omega_{1}\right|d\omega_{1}d\omega_{2}d\theta , \end{array}$$

with $\vec{k}=\omega_{1}{\vec{e}}_{1}(\theta )+\omega_{2}{\vec{e}}_{2}(\theta ).$ In [1], we obtained the absolute value of the Jacobian, |*ω*_1_| for the coordinate transform from (*k*_1_, *k*_2_, *k*_3_) to (*ω*_1_, *ω*_2_, *θ*), by similarity between the Γ coordinate system and the cylindrical system. 

 Based on (29) in the Γ coordinate system, the absolute value of the Jacobian factor can be calculated as follows:

(33) $$\begin{array}{cc} \left|\left(\frac{\partial \vec{k}}{\partial \omega_{1}}\times \frac{\partial \vec{k}}{\partial \omega_{2}}\right)\cdot \frac{\partial \vec{k}}{\partial \theta }\right| & =\left|\left({\vec{e}}_{1}\times {\vec{e}}_{2}\right)\cdot \left(\omega_{1}\frac{d}{d\theta }{\vec{e}}_{1}+\omega_{2}\frac{d}{d\theta }{\vec{e}}_{2}\right)\right|\\ & =\left|{\vec{e}}_{3}\cdot \left(\omega_{1}\frac{d}{d\theta }{\vec{e}}_{1}+\omega_{2}\frac{d}{d\theta }{\vec{e}}_{2}\right)\right|\\ & =\left|\omega_{1}\right|, \end{array}$$

where we have used the relations discussed in the next section

(34) $$\begin{array}{cc} & {\vec{e}}_{3}\cdot \frac{d}{d\theta }{\vec{e}}_{1}=-1,\;\;{\vec{e}}_{3}\cdot \frac{d}{d\theta }{\vec{e}}_{2}=0. \end{array}$$

Then, a weight function *W*(*ω*_1_, *ω*_2_, *θ*) is defined for all the coordinates (*ω*_1_, *ω*_2_, *θ*), such that the summation of the weight function on the $J(\vec{k})$ groups of coordinates of the same $\vec{k}\in R^{3}$ remains one, that is,

(35) ∑j=1J(k⃗)W(ω1  j,ω2  j,θ  j)=1.

An example of the weight functions is [1, 8]

(36) $$\begin{array}{cc} & W\left(\omega_{1},\omega_{2},\theta \right)=\mathrm{sgn}\;\left(k_{2}\right)\mathrm{sgn}\;\left(\omega_{1}\right), \end{array}$$

with $k_{2}=\vec{k}\cdot \vec{j}=\left[\omega_{1}{\vec{e}}_{1}\left(\theta \right)+\omega_{2}{\vec{e}}_{2}\left(\theta \right)\right]\cdot \vec{j}.$

 With this weight function, (32) becomes a parallel-beam reconstruction formula from the Fourier slice theorem, and further Ye-Wang's cone-beam formula [8] via the relationship between parallel-beam and cone-beam projections [1]. With the weight function, the filtration is no longer along the tangential direction generally. 

Clearly, the weight function is not unique. For instance, for frequency points with $J\left(\vec{k}\right)=3,$ in addition to the weight function defined by (36), (1 − 1 + 1), we can also choose (1/3+ 1/3+1/3), (0+1+0), or a more complicated one, (1 + 1 − 1) for some points and (−1 + 1 + 1) for the rest points, leading to different parallel-beam reconstruction formulas. Furthermore, using the approach described in [1], we can arrive at Katsevich's helical cone-beam formulas [9–11], and so forth. 

If one takes the weight function *W*(*ω*_1_, *ω*_2_, *θ*) = 1 on the right side of (32), what we will reconstruct is actually another function

(37) $$\begin{array}{cc} \Phi \left(\vec{r}\right) & =\overset{\theta_{0}}{\underset{0}{\int }}\overset{\infty }{\underset{-\infty }{\int }}\overset{\infty }{\underset{-\infty }{\int }}\exp\;\left(2\pi i\vec{k}\cdot \vec{r}\right)\hat{\Psi }\left(\vec{k}\right)\left|\omega_{1}\right|d\omega_{1}d\omega_{2}d\theta \\ & =\overset{}{\underset{R^{3}}{\int }}\exp\;\left(2\pi i\vec{k}\cdot \vec{r}\right)\hat{\Psi }\left(\vec{k}\right)J\left(\vec{k}\right)d^{3}\vec{k}. \end{array}$$

From (37), we observe that the reconstructed image $\Phi (\vec{r})$ equals the true object $\Psi (\vec{r})$ if and only if $J(\vec{k})=1,$ that is, the trajectory Γ_AB_ is a great semicircle. The condition for $\Phi (\vec{r})$ to be an approximation of the object function, that is, $\Phi (\vec{r})\approx \Psi (\vec{r}),$ is that $J\left(\vec{k}\right)=1$ in a major portion of the Fourier domain and $J\left(\vec{k}\right)$ is close to 1 in the rest region. Based on the relation between $J\left(\vec{k}\right)$ and the curve length of Γ_AB_, a necessary condition is *θ*_0_ ≈ *π*, or equivalently *ε* = *θ*_0_/*π* − 1 ≈ 0. Otherwise, $\Phi (\vec{r})$ and $\Psi (\vec{r})\;$will have a great difference. Unfortunately this formula was once thought as exact, which was essentially Theorem 3 in [2] and Theorem 4.3 in [3]. The details can be seen in [1]. 

Generally speaking, the criterion to identify exact formulas from approximate ones is to evaluate if every frequency component contributes exactly once to the final reconstruction, and the difference between exact formulas comes from different forms of weight functions. 

## 4. Motion of the Frequency Plane and Condition for Sharp Points

In this section, we will study motion of the frequency plane, present the condition for sharp points of the locus of the rotation axis, and introduce the Euler angles. Let us simplify notations ${\dot{\vec{e}}}_{3}=\left(d/d\theta \right){\vec{e}}_{3},{\ddot{\vec{e}}}_{3}=\left(d^{2}/d\theta^{2}\right){\vec{e}}_{3},$ and so on. In this section, we assume that Γ is a three times differentiable curve on the unit ball, which may be not complete. 

Obviously we have the following.

**Proposition 1**

For any point *S*(*θ*) ∈ Γ, ${\vec{e}}_{1},{\vec{e}}_{2},{\vec{e}}_{3}$ defined by (27) are orthogonal unit vectors.

**Definition 1**

For every point *S*(*θ*) ∈ Γ, let us define

(38) $$\begin{array}{cc} & \sigma ={\vec{e}}_{3}\cdot \left({\dot{\vec{e}}}_{3}\times {\ddot{\vec{e}}}_{3}\right), \end{array}$$

which is the signed volume spanned by the three vectors ${\vec{e}}_{3},{\dot{\vec{e}}}_{3},{\ddot{\vec{e}}}_{3}$. If we consider the curve length *θ* as time, ${\vec{e}}_{3},{\dot{\vec{e}}}_{3},{\ddot{\vec{e}}}_{3}$ are the position, velocity, and the acceleration of point S.

**Proposition 2**

The motion of ${\vec{e}}_{1},{\vec{e}}_{2},{\vec{e}}_{3}$ can be described by the following differential equations

(39) $$\begin{array}{c} \frac{d}{d\theta }{\vec{e}}_{3}={\vec{e}}_{1},\\ \frac{d}{d\theta }{\vec{e}}_{1}=\sigma {\vec{e}}_{2}-{\vec{e}}_{3},\\ \frac{d}{d\theta }{\vec{e}}_{2}=-\sigma {\vec{e}}_{1}. \end{array}$$

**Proof**

The first equation is the definition. Now, let us prove the other two. Since ${\vec{e}}_{1},{\vec{e}}_{2},{\vec{e}}_{3}$ are orthogonal unit vectors, we can write

(40) $$\begin{array}{c} {\vec{e}}_{1}\cdot {\vec{e}}_{1}=1,\;{\vec{e}}_{2}\cdot {\vec{e}}_{2}=1,\;{\vec{e}}_{3}\cdot {\vec{e}}_{3}=1,\\ {\vec{e}}_{1}\cdot {\vec{e}}_{2}=0,\;{\vec{e}}_{2}\cdot {\vec{e}}_{3}=0,\;{\vec{e}}_{3}\cdot {\vec{e}}_{1}=0. \end{array}$$

Applying *d*/*dθ* on the above equations, one has

(41) $$\begin{array}{c} {\dot{\vec{e}}}_{1}\cdot {\vec{e}}_{1}=0,\;{\dot{\vec{e}}}_{2}\cdot {\vec{e}}_{2}=0,\;{\dot{\vec{e}}}_{3}\cdot {\vec{e}}_{3}=0,\\ {\dot{\vec{e}}}_{1}\cdot {\vec{e}}_{2}+{\vec{e}}_{1}\cdot {\dot{\vec{e}}}_{2}=0,\;{\dot{\vec{e}}}_{2}\cdot {\vec{e}}_{3}=0,\;{\vec{e}}_{3}\cdot {\dot{\vec{\text{e}}}}_{1}=-1. \end{array}$$

Using the definition of *σ*, we have

(42) $$\begin{array}{c} {\dot{\vec{e}}}_{1}\cdot {\vec{e}}_{2}={\dot{\vec{e}}}_{1}\cdot \left({\vec{e}}_{3}\times {\vec{e}}_{1}\right)={\vec{e}}_{3}\cdot \left({\dot{\vec{e}}}_{3}\times {\ddot{\vec{e}}}_{3}\right)=\sigma ,\\ {\vec{e}}_{1}\cdot {\dot{\vec{e}}}_{2}=-\sigma . \end{array}$$

Using the orthogonal unit vectors ${\vec{e}}_{1},{\vec{e}}_{2},{\vec{e}}_{3}$, ${\dot{\vec{e}}}_{1}$ and ${\dot{\vec{e}}}_{2}$ can be expressed as

(43) $$\begin{array}{c} \frac{d}{d\theta }{\vec{e}}_{1}=\sigma {\vec{e}}_{2}-{\vec{e}}_{3},\\ \frac{d}{d\theta }{\vec{e}}_{2}=-\sigma {\vec{e}}_{1}, \end{array}$$

which completes the proof.

From these differential equations, we recognize that the motion of ${\vec{e}}_{1},{\vec{e}}_{2},{\vec{e}}_{3}$ is a rotation in the 3D space. 

Now, we consider the frequency plane $\Pi (\vec{OS})$ fixed with ${\vec{e}}_{1},{\vec{e}}_{2}$, ${\vec{e}}_{3}$ as a rigid body. When *S*(*θ*) moves along the curve Γ, the rigid body rotates in the 3D space. 

Let us define

(44) $$\begin{array}{cc} & \vec{\Omega }={\vec{e}}_{2}+\sigma {\vec{e}}_{3}. \end{array}$$

We will see that $\vec{\Omega }$ is the angular velocity of the rigid body by the following proposition.

**Proposition 3**

The motion of the vectors ${\vec{e}}_{1},{\vec{e}}_{2},{\vec{e}}_{3}$ can be expressed as

(45) $$\begin{array}{c} \frac{d}{d\theta }{\vec{e}}_{3}=\vec{\Omega }\times {\vec{e}}_{3},\\ \frac{d}{d\theta }{\vec{e}}_{1}=\vec{\Omega }\times {\vec{e}}_{1},\\ \frac{d}{d\theta }{\vec{e}}_{2}=\vec{\Omega }\times {\vec{e}}_{2}. \end{array}$$

**Proof**

It is easy to verify the three equations one by one with Proposition 2 and the definition of $\vec{\Omega }$.

**Proposition 4**

The angular acceleration of the rigid body is

(46) $$\begin{array}{cc} & \dot{\vec{\Omega }}=\dot{\sigma }{\vec{e}}_{3}\left(\theta \right). \end{array}$$

**Proof**

In fact, we have

(47) $$\begin{array}{cc} \dot{\vec{\Omega }} & =\frac{d}{d\theta }\left({\vec{e}}_{2}+\sigma {\vec{e}}_{3}\right)\\ & ={\dot{\vec{e}}}_{2}+\dot{\sigma }{\vec{e}}_{3}+\sigma {\dot{\vec{e}}}_{3}\\ & =-\sigma {\vec{e}}_{1}+\dot{\sigma }{\vec{e}}_{3}+\sigma {\vec{e}}_{1}\\ & =\dot{\sigma }{\vec{e}}_{3}. \end{array}$$

**Proposition 5**

For any point *S*(*θ*) ∈ Γ, we have

(48) $$\begin{array}{cc} & \sigma =0\Leftrightarrow {\ddot{\vec{e}}}_{3}=-{\vec{e}}_{3}, \end{array}$$

(49) $$\begin{array}{cc} & \dot{\sigma }=0\Leftrightarrow {\dddot{\vec{e}}}_{3}=-\left(\sigma^{2}+1\right){\dot{\vec{e}}}_{3}. \end{array}$$

**Proof**

Let us first prove (48).

If *σ* = 0, by Proposition 2 we have ${\ddot{\vec{e}}}_{3}=-{\vec{e}}_{3}$. On the other hand, if ${\ddot{\vec{e}}}_{3}=-{\vec{e}}_{3}$, we have $\sigma ={\vec{e}}_{3}\cdot [{\dot{\vec{e}}}_{3}\times {\ddot{\vec{e}}}_{3}]=0$.

Now, let us prove (49).

By Proposition 2, we have

(50) $$\begin{array}{c} {\dot{\vec{e}}}_{1}=\sigma {\vec{e}}_{2}-{\vec{e}}_{3}. \end{array}$$

Applying *d*/*dθ* to both sides of (50) and using $\dot{\sigma }=0$, we have

(51) $$\begin{array}{cc} {\ddot{\vec{e}}}_{1} & =\dot{\sigma }{\vec{e}}_{2}+\sigma {\dot{\vec{e}}}_{2}-{\dot{\vec{e}}}_{3}\\ & =\sigma \left(-\sigma {\vec{e}}_{1}\right)-{\vec{e}}_{1}\\ & =-\left(\sigma^{2}+1\right){\vec{e}}_{1}. \end{array}$$

On the other hand, if ${\dddot{\vec{e}}}_{3}=-(\sigma^{2}+1){\dot{\vec{e}}}_{3}$, we have

(52) $$\begin{array}{cc} \dot{\sigma } & =\frac{d}{d\theta }\left[{\vec{e}}_{3}\cdot \left({\dot{\vec{e}}}_{3}\times {\ddot{\vec{e}}}_{3}\right)\right]\\ & ={\dot{\vec{e}}}_{3}\cdot \left({\dot{\vec{e}}}_{3}\times {\ddot{\vec{e}}}_{3}\right)+{\vec{e}}_{3}\cdot \left({\ddot{\vec{e}}}_{3}\times {\ddot{\vec{e}}}_{3}\right)+{\vec{e}}_{3}\cdot \left({\dot{\vec{e}}}_{3}\times {\dddot{\vec{e}}}_{3}\right)\\ & ={\vec{e}}_{3}\cdot \left({\dot{\vec{e}}}_{3}\times {\dddot{\vec{e}}}_{3}\right)=0. \end{array}$$

This completes the proof. Physically, ${\dddot{\vec{e}}}_{3}$ is the change rate of the acceleration ${\ddot{\vec{e}}}_{3}$.

**Proposition 6**

*σ* = 0 for every point *S*(*θ*) ∈ Γ, if and only if Γ is a great circle or a great arc.

**Proof**

Since *σ* = 0 for every point *S*(*θ*) ∈ Γ, the motion equation of the three unit vectors, (39), is simplified as

(53) $$\begin{array}{c} \frac{d}{d\theta }{\vec{e}}_{3}={\vec{e}}_{1},\\ \frac{d}{d\theta }{\vec{e}}_{1}=-{\vec{e}}_{3},\\ \frac{d}{d\theta }{\vec{e}}_{2}=0. \end{array}$$

**Figure 4:**
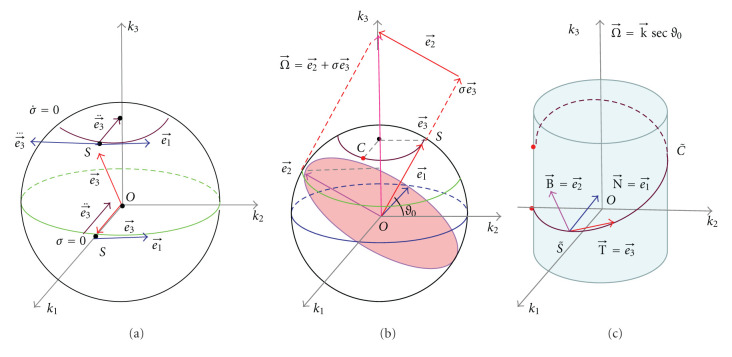
The circles on unit sphere and a helix in 3D space. (a) The illustration of *σ* = 0 and $\dot{\sigma }=0$, (b) a circular locus on the unit sphere and the motion of the frequency plane, and (c) the helix in 3D space and the motion of its TNB frame. The figure is not drawn to scale.

Since $(d/d\theta ){\vec{e}}_{2}=0$ for every point, ${\vec{e}}_{2}$ is a fixed unit vector. Since $(d/d\theta ){\vec{e}}_{3}={\vec{e}}_{1}={\vec{e}}_{2}\times {\vec{e}}_{3}$, ${\vec{e}}_{3}$ rotates about the fixed axis ${\vec{e}}_{2}$. Since ${\vec{e}}_{3}⊥{\vec{e}}_{2}$, *S*, the endpoint of ${\vec{e}}_{3}$, draws a great circle or a great arc; see Figure 4(a).

On the other hand, if Γ is a great circle or a great arc, one can verify that ${\ddot{\vec{e}}}_{3}=-{\vec{e}}_{3}.$ Therefore, we have $\sigma ={\vec{e}}_{3}\cdot ({\dot{\vec{e}}}_{3}\times {\ddot{\vec{e}}}_{3})=0$. This completes the proof.

**Proposition 7**

$\dot{\sigma }=0$ for every point *S*(*θ*) ∈ Γ, if and only if Γ is a circle or a circular arc.

**Proof**

Since $\dot{\sigma }=0,$ by Proposition 4 we have $\dot{\vec{\Omega }}=\dot{\sigma }{\vec{e}}_{3}(\theta )=0.$ We know that $\vec{\Omega }$ is a fixed vector. By Proposition 3, we have

(54) $$\begin{array}{cc} & \frac{d}{d\theta }{\vec{e}}_{3}=\vec{\Omega }\times {\vec{e}}_{3}. \end{array}$$

Therefore, ${\vec{e}}_{3}$ rotates about the fixed axis $\vec{\Omega }$, and *S*, the endpoint of ${\vec{e}}_{3}$, draws a circular arc. Furthermore, the radius of the circle is $1/\sqrt{1+\sigma^{2}}$; see Figure 4(b).

On the other hand, if Γ is a circular arc, the line connecting the circle center and the origin *O* is the rotation axis. We know that ${\ddot{\vec{e}}}_{3}$ is pointing to the axis. By circular symmetry, $\sigma ={\vec{e}}_{3}\cdot ({\dot{\vec{e}}}_{3}\times {\ddot{\vec{e}}}_{3})$ is constant at every point of the circular arc. Therefore, $\dot{\sigma }=0$ on the circular arc. This completes the proof.

Clearly, Proposition 6 is a special case of Proposition 7; see Figure 4(a).

One important case is that $\sigma ={\vec{e}}_{3}\cdot ({\dot{\vec{e}}}_{3}\times {\ddot{\vec{e}}}_{3})=0,$ for a certain point *S*(*θ*) ∈ Γ. At such a moment, we have $(d/d\theta ){\vec{e}}_{2}=-\sigma {\vec{e}}_{1}=0$, the rotation axis of the frequency plane does not move, the point *S* moves along a great arc, and the Γ coordinate system is more similar to a cylindrical coordinate system. We say that *θ* is a stationary point of ${\vec{e}}_{2}$ if *σ*(*θ*) = 0. If *σ* has different signs before and after *σ* = 0, the stationary point will become a sharp point, since in this case ${\vec{e}}_{2}$ goes forward, stops and goes back according to $(d/d\theta ){\vec{e}}_{2}=-\sigma {\vec{e}}_{1}.$ Therefore, we have the following straightforward proposition.

**Proposition 8**

A necessary condition for the curve ${\vec{e}}_{2}(\theta )$ to have a sharp point at *θ*_1_ ∈ (0, *θ*_0_) is *σ*(*θ*_1_) = 0; a sufficient condition for the curve ${\vec{e}}_{2}(\theta )$ to have a sharp point at *θ*_1_ ∈ (0, *θ*_0_) is that *σ*(*θ*) has different signs for *θ* < *θ*_1_ and for *θ* > *θ*_1_.

Based on the definition of *σ* and the equation of the osculating plane, *σ*(*θ*_1_) = 0 means that the osculating plane at *S*(*θ*_1_) ∈ Γ passes through the origin since $({\vec{e}}_{3}-0)\cdot ({\dot{\vec{e}}}_{3}\times {\ddot{\vec{e}}}_{3})=0.$  *σ*(*θ*) changes its sign before and after *θ*_1_ means that the osculating plane sweeps the origin, or relatively speaking, the origin goes from one side of the osculating plane to the other. An example can be seen in the next section. Sharp points serve as the landmarks for defining weight functions.

**Proposition 9**

For any given point on the frequency plane $\vec{k}=\omega_{1}{\vec{e}}_{1}+\omega_{2}{\vec{e}}_{2}$, the motion equation is

(55) $$\begin{array}{cc} \frac{d}{d\theta }\vec{k} & =\vec{\Omega }\times \vec{k}\\ & =-\sigma \omega_{2}{\vec{e}}_{1}\left(\theta \right)+\sigma \omega_{1}{\vec{e}}_{2}\left(\theta \right)-\omega_{1}{\vec{e}}_{3}\left(\theta \right). \end{array}$$

**Proof**

It is straightforward from Propositions 2 or  3.

We call (55) the motion equation of the frequency plane.

There are two applications of Proposition 9. First, it can be used to compute the Jacobian of the Γ coordinate system

(56) $$\begin{array}{cc} & \left|\left(\frac{\partial \vec{k}}{\partial \omega_{1}}\times \frac{\partial \vec{k}}{\partial \omega_{2}}\right)\cdot \frac{\partial \vec{k}}{\partial \theta }\right|=\left|{\vec{e}}_{3}\cdot \frac{\partial \vec{k}}{\partial \theta }\right|=\left|-\omega_{1}\right|=\left|\omega_{1}\right|. \end{array}$$

Second, since

(57) $$\begin{array}{c} {\vec{e}}_{1}\cdot \frac{\partial }{\partial \theta }\vec{k}=-\sigma \omega_{2},\\ {\vec{e}}_{2}\cdot \frac{\partial }{\partial \theta }\vec{k}=\sigma \omega_{1}, \end{array}$$

we know that among the three vectors $\partial \vec{k}/\partial \omega_{1},$$\;\partial \vec{k}/\partial \omega_{2},\;$$\partial \vec{k}/\partial \theta$, the last one is not orthogonal to the first two. Hence, a general Γ coordinate system is not orthogonal. Notice that $\partial \vec{k}/\partial \theta ={\;\partial \vec{k}/\partial \theta |}_{\omega_{1},\omega_{2}}=d\vec{k}/d\theta .$

**Figure 5:**
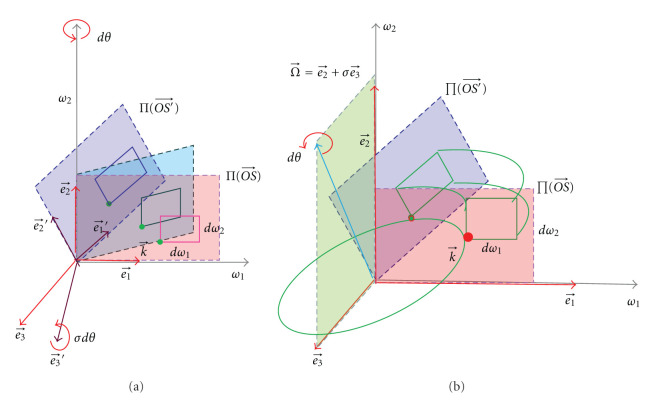
Differential motion of the frequency plane. (a) Two decomposed rotations and (b) one combined rotation.

Note that the frequency plane rotates about the axis ${\vec{e}}_{2}$ with the unit angular velocity while the vectors ${\vec{e}}_{1}$ and ${\vec{e}}_{2}$ themselves rotate about the axis ${\vec{e}}_{3}$ with the angular velocity *σ*; see Figure 5(a). We see that the Γ coordinate system is different from the cylindrical coordinate system in the motion process. However, since the spin about the axis ${\vec{e}}_{3}$ of the frequency plane does not have an effect on the scanned volume, their Jacobians have the same expression.  Figure 5(b) shows the shape of a differential volume of the Γ coordinate system.

**Proposition 10**

When S moves along a curve Γ on the unit sphere, the scanned volume in the unit ball by the frequency plane is

(58) $$\begin{array}{cc} & V=\frac{4\theta_{0}}{3}=\overset{}{\underset{\left|\vec{k}\right|<1}{\int }}J\left(\vec{k}\right)d^{3}\vec{k}, \end{array}$$

where *θ*_0_ is the length of Γ, and $J(\vec{k})$ is the number of times point $\vec{k}$ is scanned.

**Proof**

In fact, the scanned volume can be calculated in both the Γ and the Cartesian coordinate systems as follows:

(59) $$\begin{array}{cc} V & =\overset{\theta_{0}}{\underset{0}{\int }}\overset{}{\underset{k^{2}=\omega_{1}^{2}+\omega_{2}^{2}<1}{\int }}\left|\omega_{1}\right|d\omega_{1}d\omega_{2}d\theta \\ & =\overset{\theta_{0}}{\underset{0}{\int }}\overset{1}{\underset{k=0}{\int }}\overset{2\pi }{\underset{\phi =0}{\int }}k^{2}\left|\cos\;\phi \right|dkd\phi d\theta \\ & =\frac{4}{3}\overset{\theta_{0}}{\underset{0}{\int }}d\theta =\frac{4}{3}\theta_{0}\\ & =\overset{}{\underset{k_{1}^{2}+k_{2}^{2}+k_{3}^{2}<1}{\int }}J\left(\vec{k}\right)d^{3}\vec{k}. \end{array}$$

This completes the proof.

This concise formula provides a basic relation between the curve length, the scanned volume by the frequency plane, and the J function. 

**Figure 6:**
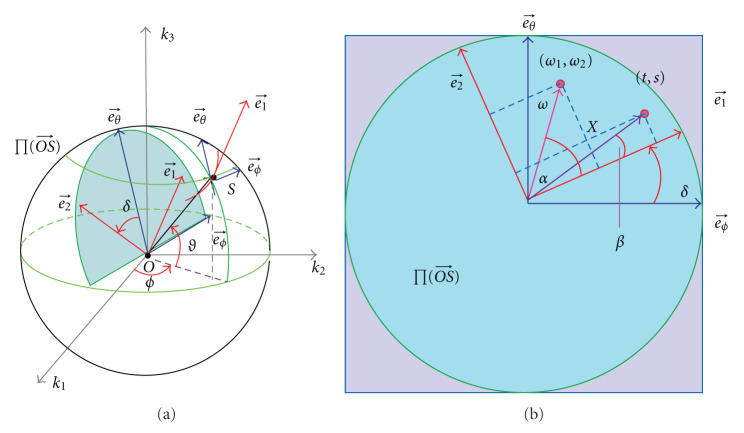
Euler angles. (a) The Euler angles of the frequency plane and (b) the rotation angle of frequency plane relative to the detector plane.

When we study the rotation problem, Euler angles (*ϑ*, *ϕ*, *δ*) are helpful. As shown in Figure 6(a), (*ϑ*, *ϕ*) with *ϑ* ∈ (−*π*/2, *π*/2), *ϕ* ∈ (−*∞*, *∞*) are the spherical coordinate of *S* = *S*(*ϑ*, *ϕ*), *δ* is the angle between ${\vec{e}}_{\vartheta }$ and ${\vec{e}}_{2}$. ${\vec{e}}_{\phi },$ and ${\vec{e}}_{\vartheta }$ are the latitude and longitude unit vectors at point *S* in the spherical coordinate system. 

The Euler angles are functions of the curve length

(60) $$\begin{array}{c} \vartheta =\vartheta \left(\theta \right),\\ \phi =\phi \left(\theta \right),\\ \delta =\delta \left(\theta \right). \end{array}$$

The three unit vectors can be represented as follows:

(61) $$\begin{array}{c} {\vec{e}}_{3}={\vec{e}}_{3}\left(\vartheta ,\phi \right),\\ {\vec{e}}_{1}=\sin\;\delta {\vec{e}}_{\vartheta }+\cos\;\delta {\vec{e}}_{\phi }=\dot{\vartheta }{\vec{e}}_{\vartheta }+\dot{\phi }\cos\;\vartheta {\vec{e}}_{\phi },\\ {\vec{e}}_{2}=-\cos\;\delta {\vec{e}}_{\vartheta }+\sin\;\delta {\vec{e}}_{\phi }=-\dot{\phi }\cos\;\vartheta {\vec{e}}_{\vartheta }+\dot{\vartheta }{\vec{e}}_{\phi }. \end{array}$$

Furthermore, one can verify the motion equations of the three unit vectors in Proposition 2. 

Based on the motion theory of rigid body, the angular velocity of a rigid body can be written as

(62) $$\begin{array}{cc} \vec{\Omega } & =-\dot{\vartheta }{\vec{e}}_{\phi }+\dot{\phi }\vec{k}+\dot{\delta }{\vec{e}}_{3}\\ & =-\dot{\vartheta }{\vec{e}}_{\phi }+\dot{\phi }\cos\;\vartheta {\vec{e}}_{\vartheta }+\left(\dot{\phi }\sin\;\vartheta +\dot{\delta }\right){\vec{e}}_{3}\\ & ={\vec{e}}_{2}+\sigma {\vec{e}}_{3}. \end{array}$$

Therefore, we have the following relationships:

(63) $$\begin{array}{c} {\vec{e}}_{2}=-\dot{\vartheta }{\vec{e}}_{\phi }+\dot{\phi }\cos\;\vartheta {\vec{e}}_{\vartheta }={\vec{\Omega }}_{0}-\left({\vec{\Omega }}_{0}\cdot {\vec{e}}_{3}\right){\vec{e}}_{3},\\ \sigma =\dot{\phi }\sin\;\vartheta +\dot{\delta }={\vec{\Omega }}_{0}\cdot {\vec{e}}_{3}-{\dot{\vec{\Omega }}}_{0}\cdot {\vec{e}}_{1},\\ \dot{\delta }=-{\dot{\vec{\Omega }}}_{0}\cdot {\vec{e}}_{1}={\vec{\Omega }}_{0}\cdot {\dot{\vec{e}}}_{1}, \end{array}$$

where we have used a new notation

(64) $$\begin{array}{cc} & {\vec{\Omega }}_{0}=-\dot{\vartheta }{\vec{e}}_{\phi }+\dot{\phi }\vec{k}, \end{array}$$

which is the first two terms of the total angular velocity $\vec{\Omega }$ of (62). 

In fact, ${\vec{\Omega }}_{0}$ is the angular velocity of the detector with the unit vectors ${\vec{e}}_{\phi },{\vec{e}}_{\vartheta }$ when *S* moves along Γ. To be specific, we consider the detector for parallel-beam projection as a rectangle of finite width and length through the origin orthogonal to direction ${\vec{e}}_{3}=\vec{OS}$. During any motion, one side of the detector always keeps horizontal, that is, parallel to the direction ${\vec{e}}_{\phi }.$ The pose of the detector is completely determined by the point *S* = *S*(*ϑ*, *ϕ*), or two Euler's angles (*ϑ*, *ϕ*). The relative motion of the frequency plane to the detector is described by the angle *δ*; see Figure 6(b). 

In summary, there are three sets of orthogonal vectors (beside $\vec{\text{i}},\vec{\text{j}},\vec{\text{k}}$, the unit vectors of the Cartesian coordinate system) and three important planes. The orthogonal unit vectors ${\vec{e}}_{\phi },{\vec{e}}_{\vartheta }$, ${\vec{e}}_{3}$ are related to the movement of the detector and ${\vec{e}}_{3}$, ${\dot{\vec{e}}}_{3}{=\vec{e}}_{1}$, ${\vec{e}}_{2}={\vec{e}}_{3}\times {\vec{e}}_{1}$ are related to the movement of the frequency plane, see Figure 6(b). It is easy to verify that $\vec{\Omega }={\vec{e}}_{2}+{\sigma \vec{e}}_{3}={\vec{e}}_{1}\times {\dot{\vec{e}}}_{1}$ and hence the vectors ${\vec{e}}_{1}$, ${\dot{\vec{e}}}_{1}$, $\vec{\Omega }$ are the third set of orthogonal vectors. The direction of $\vec{\Omega }$ is the normal of the osculating plane and its magnitude, $\sqrt{1+\sigma^{2}}$, is the reciprocal of the radius of the osculating circle. Here the osculating plane is spanned by ${\vec{e}}_{1}$, ${\dot{\vec{e}}}_{1}$ [15] and the osculating circle is referred to as the intersection between the osculating plane and the unit ball. When S moves along a curve Γ on the unit ball, it can be viewed that the point S moves along an osculating circle, whose orientation and radius are described by $\vec{\Omega }$. From $\vec{\Omega }={\vec{e}}_{2}+{\sigma \vec{e}}_{3}$, we can see that *σ* is an angular velocity of the spin of the frequency plane. The locus lengths of the endpoints of ${\vec{e}}_{3},$${\vec{e}}_{1}$, ${\vec{e}}_{2}$ are *θ*_0_, $\int_{0}^{\theta_{0}}\sqrt{1+\sigma^{2}}d\theta$, ∫_0_^*θ*_0_^|*σ*|*dθ*, respectively, according to Proposition 2.

## 5. Example of a Curve with $J(\vec{k})=1,3$

Here, we discuss the reconstruction based on the simplified model of the 3D helical cone beam reconstruction, that is, 3D helical parallel-beam reconstruction. 

**Figure 7:**
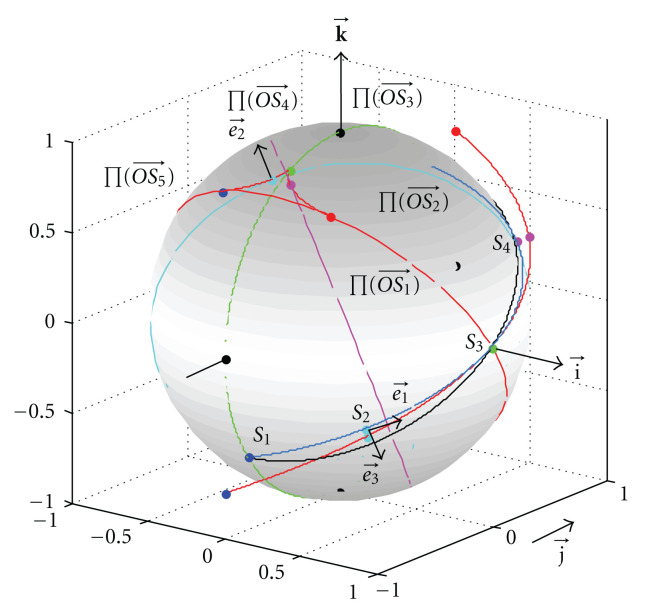
Motion process of the frequency plane. The red curve is a segment of a helical scanning trajectory. The blue curve is its projection on the unit sphere. The black curve is the great circle through *S*_1_  , *S*_3_, and *S*_5_. Five positions of the frequency plane are color coded. The points inside the triangle are scanned 3 times while the rest points are scanned only once.

The curve *C* in *R*^3^ defined by

(65) $$\begin{array}{cc} C & =\{S^{\prime }\in R^{3}:\vec{OS^{\prime }}=\vec{y}\left(\varphi \right)=\left(r\cos\;\varphi ,r\sin\;\varphi ,\varphi \frac{h}{2\pi }\right),\;\\ & \;\;\;\varphi \in \left[-\frac{\pi }{2},\frac{\pi }{2}\right]\} \end{array}$$

is a segment of a helix with radius *r* and pitch *h*, which is shown in Figure 7 for *r* = 1, *h* = 3.

 In fact, it is formed by a combination of a uniform circular motion in the XOY plane and a uniform straight motion along the *Z* direction. 

The projection of the curve *C* on the unit sphere Ω is

(66) $$\begin{array}{cc} & \Gamma =\left\{S\in \Omega :\vec{OS}={\vec{e}}_{3}\left(\varphi \right)=\frac{\vec{y}\left(\varphi \right)}{\left|\vec{y}\left(\varphi \right)\right|},\varphi \in \left[-\frac{\pi }{2},\frac{\pi }{2}\right]\right\}, \end{array}$$

or

(67) $$\begin{array}{cc} & \Gamma =\left\{S\in \Omega :\vec{OS}={\vec{e}}_{3}\left(\vartheta ,\phi \right),\vartheta =\text{t}{\text{g}}^{-1}\left(\frac{h\phi }{2\pi }\right),\phi \in \left[-\frac{\pi }{2},\frac{\pi }{2}\right]\right\}. \end{array}$$

The curve length *θ* can be given by

(68) $$\begin{array}{c} d\theta =\left|d{\vec{e}}_{3}\left(\varphi \right)\right|=\left|d\left(\frac{\vec{y}\left(\varphi \right)}{\left|\vec{y}\left(\varphi \right)\right|}\right)\right|,\\ \theta \left(\varphi \right)=\overset{\varphi }{\underset{-\pi /2}{\int }}\frac{d\theta }{d\varphi^{\prime }}d\varphi^{\prime }=\overset{\varphi }{\underset{-\pi /2}{\int }}\left|d\left(\frac{\vec{y}\left(\varphi^{\prime }\right)}{\left|\vec{y}\left(\varphi^{\prime }\right)\right|}\right)\right|. \end{array}$$

For example, we can calculate *θ*_0_ = *θ*(*π*/2) ≈ 3.177 and *ε* = 0.011, when *r* = 1 and *h* = 3, indicating that the overlapping degree in the Fourier domain is small. 

For every *φ* ∈ [−*π*/2, *π*/2], the three orthogonal unit vectors are

(69) $$\begin{array}{c} {\vec{e}}_{3}=\vec{OS}=\frac{\vec{y}\left(\varphi \right)}{\left|\vec{y}\left(\varphi \right)\right|},\\ {\vec{e}}_{1}=\frac{\left(d/d\varphi \right){\vec{e}}_{3}}{\left|\left(d/d\varphi \right){\vec{e}}_{3}\right|},\\ {\vec{e}}_{2}={\vec{e}}_{3}\times {\vec{e}}_{1}. \end{array}$$

The inverse Fourier transform can be expressed with either the Γ coordinates (*ω*_1_, *ω*_2_, *θ*) or the new coordinates (*ω*_1_, *ω*_2_, *φ*)

(70) $$\begin{array}{cc} \Psi \left(\vec{r}\right) & =\overset{\theta_{0}}{\underset{0}{\int }}\overset{\infty }{\underset{-\infty }{\int }}\overset{\infty }{\underset{-\infty }{\int }}W\left(\omega_{1},\omega_{2},\theta \right)\exp\;\left(2\pi i\vec{k}\cdot \vec{r}\right)\hat{\Psi }\left(\vec{k}\right)\\ & \;\times \left|\omega_{1}\right|d\omega_{1}d\omega_{2}d\theta \\ & =\overset{\pi /2}{\underset{-\pi /2}{\int }}\overset{\infty }{\underset{-\infty }{\int }}\overset{\infty }{\underset{-\infty }{\int }}W\left(\omega_{1},\omega_{2},\theta \left(\varphi \right)\right)\exp\;\left(2\pi i\vec{k}\cdot \vec{r}\right)\\ & \;\times \hat{\Psi }\left(\vec{k}\right)\left|\omega_{1}\right|\frac{d\theta }{d\varphi }d\omega_{1}d\omega_{2}d\varphi , \end{array}$$

with

(71) $$\begin{array}{cc} & \vec{k}=\vec{k}\left(\omega_{1},\omega_{2},\theta \left(\varphi \right)\right)=\omega_{1}{\vec{e}}_{1}\left(\theta \left(\varphi \right)\right)+\omega_{2}{\vec{e}}_{2}\left(\theta \left(\varphi \right)\right). \end{array}$$

To visualize the motion of the frequency plane $\prod (\vec{OS})$, its five positions are marked in Figure 7 at *φ*_1_ = −*π*/2, *φ*_2_ = −*π*/4, *φ*_3_ = 0, *φ*_4_ = *π*/4 and *φ*_5_ = *π*/2, respectively. Note that the initial position $\Pi (\vec{OS_{1}})$ and the final position $\Pi (\vec{OS_{5}})$ coincide with each other. 

 In Figure 7, the two curved sides of the red triangle on the top of the unit sphere is the locus of ${\vec{e}}_{3}$, the instantaneous rotation axis of the frequency plane. Similarly, $-{\vec{e}}_{2}$ and $\Pi (\vec{OS_{1}})$ form the counterpart triangle on the bottom of the unit sphere. The locus of ${\vec{e}}_{2}$ is the division line of the regions with different $J(\vec{k})$ values. Based on (58), the total area of the two triangles is only 0.55% of the surface area of the unit sphere. 

Note that a sharp point on the locus of ${\vec{e}}_{2}$ appears at *φ* = *φ*_3_ = 0. The reason is as follows.

Let us define

(72) $$\begin{array}{cc} & \sigma_{0}\left(\varphi \right)=\vec{y}\left(\varphi \right)\cdot \left(\frac{d}{d\varphi }\vec{y}\left(\varphi \right)\times \frac{d^{2}}{d\varphi^{2}}\vec{y}\left(\varphi \right)\right). \end{array}$$

It can be verified that *σ*_0_(0) = 0, *σ*_0_(*φ*) < 0 for *φ* < 0, and *σ*_0_(*φ*) > 0 for *φ* > 0.

It is not difficult to prove

(73) $$\begin{array}{cc} \sigma & ={\vec{e}}_{3}\cdot \left({\vec{e}}_{1}\times {\dot{\vec{e}}}_{1}\right)\\ & =\vec{y}\left(\varphi \right)\cdot \left(\frac{d}{d\varphi }\vec{y}\left(\varphi \right)\times \frac{d^{2}}{d\varphi^{2}}\vec{y}\left(\varphi \right)\right)\frac{1}{\left|\vec{y}\left(\varphi \right)\right|}\;\\ & \;\times \frac{1}{{\left|\left(d/d\varphi \right)\vec{y}\left(\varphi \right)\right|}^{2}}{\left(\frac{d\varphi }{d\theta }\right)}^{3}\\ & =\sigma_{0}\left(\varphi \right)K\left(\varphi \right), \end{array}$$

where *K*(*φ*) is positive. Therefore, *σ* and *σ*_0_ have the same zeros and sign. 

Based on the motion process of $\;\Pi (\vec{OS})$, we have

(74) $$J\left(\vec{k}\right)=\{\begin{array}{cc} 1, & \vec{k}\;\text{outside}\;\text{the}\;\text{two}\;\text{triangles},\\ 3, & \vec{k}\;\text{inside}\;\text{the}\;\text{two}\;\text{triangles}. \end{array}$$

The overlapped region in the frequency space is due to the motion of the instantaneous rotation axis ${\vec{e}}_{2}$ of the frequency plane, or equivalently the discrepancy between the curve Γ and half a great circle. 

**Figure 8:**
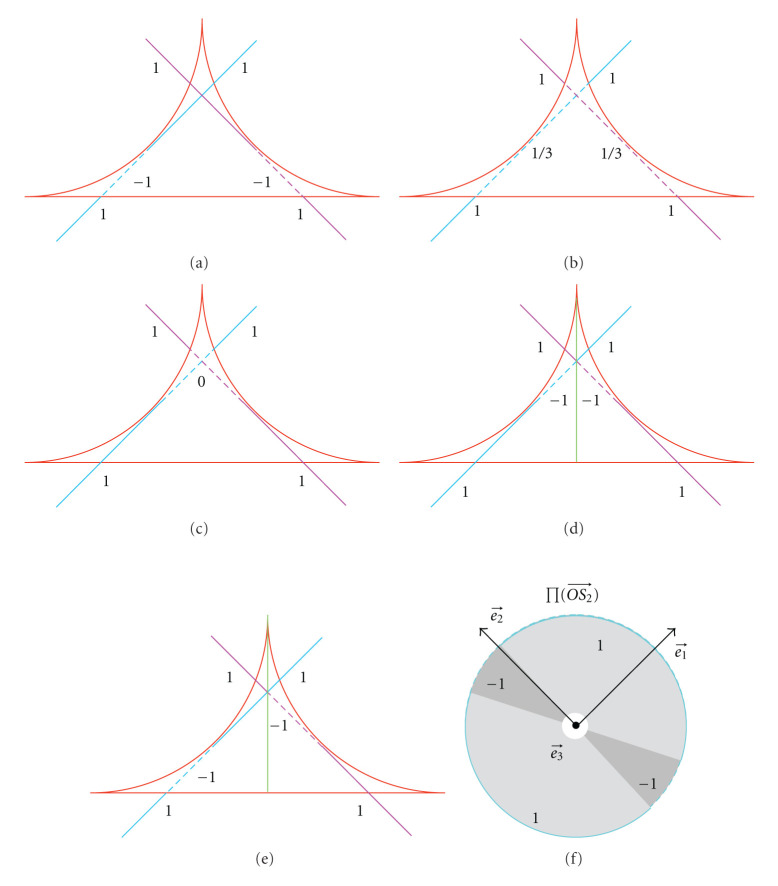
Examples of possible weight factors. (a) 1 − 1 + 1, (b) 1/3+1/3+1/3, (c) 0+1+0, and (d) −1 + 1 + 1 for the left half, 1 + 1 − 1 for the right half, (e) 1 − 1 + 1 for the left half, and 1 + 1 − 1 for the right half. The straight line represents a great circle on the unit sphere. The weight factor in (a) for $\prod \left(\vec{{OS}_{2}}\right)$ is further illustrated in (f) as an example.

As shown in Figure 8, the weight factor can be (1 − 1 + 1), (1/3+ 1/3+1/3), or (0+1+0), *and so forth*, for the overlapped region. By dividing the overlapped region into 2 symmetric parts, one can define the weight function as (−1 + 1 + 1) on the left side and (1 + 1 − 1) on the right side, or (1 − 1 + 1) on the left and (1 + 1 − 1) on the right, *and so forth*. Hence, one can design various parallel-beam reconstruction formulas and derive the associated cone-beam formulas using the approach described in [1].

## Appendices

### A. Weighted Λ Reconstruction Formula

*f*(*x*, *y*) is a function in the 2D space with the Fourier transform

(A.1)
$$\begin{array}{cc} & \hat{f}\left(k_{1},k_{2}\right)=\overset{\infty }{\underset{-\infty }{\int }}\overset{\infty }{\underset{-\infty }{\int }}f\left(x,y\right)\exp\;\left(-i2\pi k_{1}x\right)\exp\;\left(-i2\pi k_{2}y\right)dxdy. \end{array}$$

Its parallel-beam projection is denoted as

(A.2)
$$\begin{array}{cc} & P_{\vartheta }\left(l\right)=\overset{\infty }{\underset{-\infty }{\int }}f\left(l\cos\;\vartheta -v\sin\;\vartheta ,l\sin\;\vartheta +v\cos\;\vartheta \right)dv \end{array}$$

with *l* ∈ (−*∞*, *∞*), *ϑ* ∈ [−*π*/2, *π*/2].

Let *f*(*x*, *y*) pass through the filter |*k*_1_|, we get a new function

(A.3)
$$\begin{array}{cc} & \Phi \left(x,y\right)\\ & =\overset{\infty }{\underset{-\infty }{\int }}\overset{\infty }{\underset{-\infty }{\int }}\left|k_{1}\right|\hat{f}\left(k_{1},k_{2}\right)\exp\;\left(i2\pi k_{1}x\right)\exp\;\left(i2\pi k_{2}y\right)dk_{1}dk_{2}\\ & =f\left(x,y\right)*\frac{1}{-2\pi^{2}x^{2}}. \end{array}$$

Defining the signed polar coordinates (*k*_*ρ*_, *ϑ*) with *k*_*ρ*_ ∈ (−*∞*, *∞*), *ϑ* ∈ (−*π*/2, *π*/2), which satisfy *k*_1_ = *k*_*ρ*_cos *ϑ*, *k*_2_ = *k*_*ρ*_sin *ϑ*, we have

(A.4)
$$\begin{array}{cc} \Phi \left(x,y\right) & =\overset{\pi /2}{\underset{-\pi /2}{\int }}\overset{\infty }{\underset{-\infty }{\int }}\left|k_{1}\right|\hat{f}\left(k_{\rho },\vartheta \right)\\ & \;\;\;\;\times \exp\;\left[-i2\pi k_{\rho }\left(x\cos\;\vartheta +y\sin\;\vartheta \right)\right]\left|k_{\rho }\right|dk_{\rho }d\vartheta \\ & =\overset{\pi /2}{\underset{-\pi /2}{\int }}\overset{\infty }{\underset{-\infty }{\int }}\hat{f}\left(k_{\rho },\vartheta \right)\exp\;\left[-i2\pi k_{\rho }\left(x\cos\;\vartheta +y\sin\;\vartheta \right)\right]\\ & \;\;\;\;\;\;\times k_{\rho }^{2}\cos\;\vartheta dk_{\rho }d\vartheta \\ & =\frac{1}{-4\pi^{2}}\overset{\pi /2}{\underset{-\pi /2}{\int }}{\;\frac{\partial^{2}}{\partial l^{2}}|}_{l=x\cos\;\vartheta +y\sin\;\vartheta }\overset{\infty }{\underset{-\infty }{\int }}\hat{f}\left(k_{\rho },\vartheta \right)\\ & \;\;\;\;\times \exp\;\left(-i2\pi k_{\rho }l\right)dk_{\rho }\cos\;\vartheta d\vartheta \\ & =\frac{1}{-4\pi^{2}}\overset{\pi /2}{\underset{-\pi /2}{\int }}{\;\frac{\partial^{2}}{\partial l^{2}}|}_{l=x\cos\;\vartheta +y\sin\;\vartheta }P_{\vartheta }\left(l\right)\cos\;\vartheta d\vartheta . \end{array}$$

Comparing (A.3) and (A.4), we have

(A.5)
$$\begin{array}{cc} & f\left(x,y\right)*\frac{1}{-2\pi^{2}x^{2}}\\ & \;=\frac{1}{-4\pi^{2}}\overset{\pi /2}{\underset{-\pi /2}{\int }}{\;\frac{\partial^{2}}{\partial l^{2}}|}_{l=x\cos\;\vartheta +y\sin\;\vartheta }P_{\vartheta }\left(l\right)\cos\;\vartheta d\vartheta . \end{array}$$

That is, we have rediscovered the identity (20) in a general way. In contrast to the classical Λ reconstruction formula [1], a weight factor cos *ϑ* is involved in (A.5). Hence, we can call (A.5) a weighted Λ reconstruction formula.

### B. A Weighted Radon Formula and the Orlov Formula

Let us define

(B.1)
$$\begin{array}{cc} & u=\vec{r}\cdot {\vec{e}}_{3},\;t=\vec{r}\cdot {\vec{e}}_{1},\;s=\vec{r}\cdot {\vec{e}}_{2}, \end{array}$$

and the parallel-beam projection is

(B.2)
$$\begin{array}{cc} & P_{\theta }\left(t,s\right)=\overset{\infty }{\underset{-\infty }{\int }}\Psi \left(u{\vec{e}}_{3}+t{\vec{e}}_{1}+s{\vec{e}}_{2}\right)du, \end{array}$$

as shown in Figure 6(b). In the frequency plane $\Pi (\vec{OS})$, we define a signed polar coordinate system (*ω*, *α*) with *ω* ∈ (−*∞*, *∞*), *α* ∈ [0, *π*] for a point (*ω*_1_, *ω*_2_) with *ω*_1_ = *ω*cos *α*, *ω*_2_ = *ω*cos *α*. Similarly, let us define the signed polar coordinates (*X*, *β*) with *X* ∈ (−*∞*, *∞*), *β* ∈ [−*π*/2, *π*/2] for a point (*t*, *s*) with *t* = *X*cos *β*, *s* = *X*sin *β*.

Let the weight function in (32) be *W*(*ω*_1_, *ω*_2_, *θ*) = *W*(*α*, *θ*), we have

(B.3)
$$\begin{array}{cc} & \Psi \left(\vec{r}\right)\\ & =\overset{\theta_{0}}{\underset{0}{\int }}\overset{\infty }{\underset{-\infty }{\int }}\overset{\infty }{\underset{-\infty }{\int }}W\left(\omega_{1},\omega_{2},\alpha \right)\exp\;\left(2\pi i\vec{k}\cdot \vec{r}\right)\hat{\Psi }\left(\vec{k}\right)\\ & \;\;\;\;\;\;\times \left|\omega_{1}\right|d\omega_{1}d\omega_{2}d\theta \\ & =\overset{\theta_{0}}{\underset{0}{\int }}\overset{\pi }{\underset{0}{\int }}\overset{\infty }{\underset{-\infty }{\int }}W\left(\alpha ,\theta \right)\left|\cos\;\alpha \right|\exp\;\left(2\pi i\omega_{1}t+2\pi i\omega_{2}s\right)\\ & \;\;\;\;\;\times \hat{\Psi }\left(\omega_{1},\omega_{2},\theta \right)\omega^{2}d\omega d\alpha d\theta \\ & =\overset{\theta_{0}}{\underset{0}{\int }}\overset{\pi }{\underset{0}{\int }}W\left(\alpha ,\theta \right)\left|\cos\;\alpha \right|\overset{\infty }{\underset{-\infty }{\int }}\omega^{2}\exp\;\left(2\pi i\omega \left(t\cos\;\alpha +s\sin\;\alpha \right)\right)\\ & \;\;\;\;\;\;\;\;\;\;\;\times \hat{\Psi }\left(\omega ,\alpha ,\theta \right)d\omega d\alpha d\theta \\ & =\frac{-1}{4\pi^{2}}\overset{\theta_{0}}{\underset{0}{\int }}\overset{\pi }{\underset{0}{\int }}W\left(\alpha ,\theta \right){\;\frac{\partial^{2}}{\partial l^{2}}|}_{\begin{array}{c} l=\vec{r}\cdot \vec{n}\\ =t\cos\;\alpha +s\sin\;\alpha \end{array}}R\Psi \left(l,\vec{n}\right)\\ & \;\;\;\;\;\times \left|\cos\;\alpha \right|d\alpha d\theta \\ & =\frac{-1}{4\pi^{2}}\nabla^{2}\overset{\theta_{0}}{\underset{0}{\int }}\overset{\pi }{\underset{0}{\int }}W\left(\alpha ,\theta \right)R\Psi \left(t\cos\;\alpha +s\sin\;\alpha ,\vec{n}\right)\\ & \;\;\;\;\;\;\times \left|\cos\;\alpha \right|d\alpha d\theta , \end{array}$$

with $\vec{n}={\vec{e}}_{1}\cos\;\alpha +{\vec{e}}_{2}\sin\;\alpha$.

We call it the weighted Radon formula. When Γ_AB_ is a great semicircle, we have *W*(*α*, *θ*) = 1 and *θ*_0_ = *π*, and the above formula becomes the classic Radon formula [12, 13].

Moreover, it is easy to rederive Orlov's formula [14] from (32)

(B.4)
$$\begin{array}{cc} \Psi \left(\vec{r}\right) & =\overset{\theta_{0}}{\underset{0}{\int }}\overset{\infty }{\underset{-\infty }{\int }}\overset{\infty }{\underset{-\infty }{\int }}W\left(\omega_{1},\omega_{2},\theta \right)\exp\;\left(2\pi i\vec{k}\cdot \vec{r}\right)\hat{\Psi }\left(\vec{k}\right)\\ & \;\;\;\;\;\;\times \left|\omega_{1}\right|d\omega_{1}d\omega_{2}d\theta \\ & =\overset{\theta_{0}}{\underset{0}{\int }}\overset{\infty }{\underset{-\infty }{\int }}\overset{\infty }{\underset{-\infty }{\int }}W\left(\alpha ,\theta \right)\exp\;\left(2\pi i\omega_{1}t+2\pi i\omega_{2}s\right)\\ & \;\;\;\;\;\;\times \hat{\Psi }\left(\omega_{1},\omega_{2},\theta \right)\left|\omega_{1}\right|d\omega_{1}d\omega_{2}d\theta \\ & =\overset{\theta_{0}}{\underset{0}{\int }}\overset{\infty }{\underset{-\infty }{\int }}\overset{\infty }{\underset{-\infty }{\int }}\frac{-1}{4\pi^{2}}\left(\frac{\partial^{2}}{\partial t^{2}}+\frac{\partial^{2}}{\partial s^{2}}\right)W\left(\alpha ,\theta \right)\\ & \;\times \exp\;\left(2\pi i\omega_{1}t+2\pi i\omega_{2}s\right)\hat{\Psi }\left(\omega_{1},\omega_{2},\theta \right)\frac{\left|\omega_{1}\right|}{\omega^{2}}d\omega_{1}d\omega_{2}d\theta \\ & =-\frac{1}{4\pi^{2}}\nabla^{2}\overset{\theta_{0}}{\underset{0}{\int }}\overset{\infty }{\underset{-\infty }{\int }}\overset{\infty }{\underset{-\infty }{\int }}\frac{\left|\cos\;\alpha \right|}{\left|\omega \right|}W\left(\alpha ,\theta \right)\cdot \hat{\Psi }\left(\omega_{1},\omega_{2},\theta \right)\\ & \;\;\;\;\;\;\;\;\times \exp\;\left(2\pi i\omega_{1}t+2\pi i\omega_{2}s\right)d\omega_{1}d\omega_{2}d\theta \\ & =-\frac{1}{4\pi^{2}}\nabla^{2}\overset{\theta_{0}}{\underset{0}{\int }}p_{\theta }\left(t,s\right){}^{**}g_{\theta }\left(t,s\right)d\theta , \end{array}$$

where

(B.5)
$$\begin{array}{cc} & g_{\theta }\left(t,s\right)\\ & \;=\overset{\infty }{\underset{-\infty }{\int }}\overset{\infty }{\underset{-\infty }{\int }}\frac{\left|\cos\;\alpha \right|}{\left|\omega \right|}W\left(\alpha ,\theta \right)\exp\;\left(2\pi i\omega_{1}t+2\pi i\omega_{2}s\right)d\omega_{1}d\omega_{2}\\ & \;=\frac{\left|\cos\;\alpha \right|}{\left|X\right|}{\;W\left(\alpha ,\theta \right)|}_{\alpha =\pi /2+\beta }. \end{array}$$

The sign ∗∗ denotes the 2D convolution operation.

We recall that a frequency point has $J(\vec{\text{k}})$ sets of Γ coordinates

(B.6)
k⃗=k⃗(ω1,  1ω2,  1θ  1)=k⃗(ω1,  2ω2  2,θ  2)=⋯=k⃗(ω1,  J(k⃗)ω2,  J(k⃗)θ  J(k⃗)).

If a given triplet (*ω*_1_, *ω*_2_, *θ*) is the *j*th set of Γ coordinates of $\vec{k}$,

(B.7) (ω1,ω2,θ)=(ω1,  jω2  j,θ  j),

a new normalized weight function [1] for the triplet can be defined by

(B.8)
W(ω1,ω2,θ)=W(α,θ)=1/|k⃗·e⃗1(θ  j)|∑j=1J(k⃗)1/|k⃗·e⃗1(θ  j)|=1/|cos α|∑j=1J(k⃗0)1/|k⃗0·e⃗1(θ  j)|,

and in this case we have

(B.9)
gθ(t,s)=1|X| 1∑j=1J(k⃗0)1/|k⃗0·e⃗1(θ  j)||k⃗0=e⃗3×X⃗0.

Here, ${\vec{k}}^{0}=\vec{k}/|\vec{k}|$, and ${\vec{X}}^{0}=(t{\vec{e}}_{1}+s{\vec{e}}_{2})/\sqrt{t^{2}+s^{2}}={\vec{e}}_{1}\cos\;\beta +{\vec{e}}_{2}\sin\;\beta$ are unit vectors.

Equation (B.4) with (B.9) is the Orlov formula for a general curve on the unit sphere ((2a) with (2c) in [14]). Note that in [14] different letters were used such as $m↔{\vec{k}}^{0},$ $\;\tau ↔{\vec{e}}_{3},$ $\;\dot{\tau }↔{\vec{e}}_{1},$ $\;x^{\prime }↔\left(t{\vec{e}}_{1}+s{\vec{e}}_{2}\right),$ $\;\left|x^{\prime }\right|↔\left|X\right|,$ $\int_{G}^{}dG_{\tau }↔\int_{0}^{\theta_{0}}d\theta ,$ and so forth, and ${\dot{\tau }}_{k}^{\prime }$ should be printed as ${\dot{\tau }}_{k}^{}$.

### C. Link between the Motion of the Frequency Plane and Differential Geometry

To study the property of a curve Γ on the unit sphere and the associated motion of the frequency plane, one can introduce another curve, $\tilde{\Gamma }$, defined as

(C.1)
$$\begin{array}{cc} & \vec{r}\left(\theta \right)=\overset{\theta }{\underset{0}{\int }}{\vec{e}}_{3}\left(\theta^{\prime }\right)d\theta^{\prime }+\vec{r}\left(0\right), \end{array}$$

where $\vec{r}\left(0\right)$ is an arbitrary vector in the 3D space, and *θ* (as well as *θ*′) is an arc-length parameter of the curve Γ. Clearly,

(C.2) $$\begin{array}{cc} & \dot{\vec{r}}=\frac{d}{d\theta }\vec{r}\left(\theta \right)={\vec{e}}_{3},\;\;\ddot{\vec{r}}={\vec{e}}_{1},\;\;\dddot{\vec{r}}={\dot{\vec{e}}}_{1}. \end{array}$$

Suppose that the arc-length parameter of the curve $\tilde{\Gamma }$ is *s*. The tangential direction of the curve $\tilde{\Gamma }$ is

(C.3)
$$\begin{array}{cc} & \vec{\text{T}}=\frac{d}{ds}\vec{r}=\frac{d\theta }{ds}\frac{d}{d\theta }\vec{r}\left(\theta \right)=\frac{d\theta }{ds}{\vec{e}}_{3}\left(\theta \right). \end{array}$$

Furthermore, we have

(C.4)
$$\begin{array}{c} \left|\frac{d}{ds}\vec{r}\right|=\left|\frac{d\theta }{ds}\right|\left|{\vec{e}}_{3}\left(\theta \right)\right|,\\ 1=\left|\frac{d\theta }{ds}\right|, \end{array}$$

where $\left|(d/ds)\vec{r}\right|=\left|d\vec{r}/ds\right|=\left|d\vec{r}\right|/ds=ds/ds=1$ has been used.

When the positive directions are the same, we have

(C.5)
$$\begin{array}{cc} & \frac{d\theta }{ds}=1,\\ & \theta =s+s_{0},\\ & \vec{\text{T}}={\vec{e}}_{3}\left(\theta \right). \end{array}$$

Picking up the constant *s*_0_ = 0, we have *θ* = *s*. Therefore, the symbol *θ* represents the arc length parameters of both $\tilde{\Gamma }$ and Γ. Based on the tangential direction $\vec{\text{T}}$, the norm and binormal unit vectors [15] can be defined as

(C.6)
$$\begin{array}{c} \vec{\text{N}}=\frac{\dot{\vec{\text{T}}}}{\left|\dot{\vec{\text{T}}}\right|}=\frac{{\vec{e}}_{1}}{\left|{\vec{e}}_{1}\right|}={\vec{e}}_{1},\\ \vec{\text{B}}=\vec{\text{T}}\times \vec{\text{N}}={\vec{e}}_{3}\times {\vec{e}}_{1}={\vec{e}}_{2}. \end{array}$$

The curvature *κ* and the torsion *τ* of the curve $\tilde{\Gamma }$ are

(C.7)
$$\begin{array}{c} \kappa =\left|\ddot{\vec{r}}\right|=\left|{\vec{e}}_{1}\right|=1,\\ \tau =\dot{\vec{r}}\cdot \left(\ddot{\vec{r}}\times \dddot{\vec{r}}\right)={\vec{e}}_{3}\cdot \left({\vec{e}}_{1}\times {\dot{\vec{e}}}_{1}\right)=\sigma , \end{array}$$

and the Darboux vector is

(C.8)
$$\begin{array}{cc} & \vec{\Omega }=\tau \vec{\text{T}}+\kappa \vec{\text{B}}={\vec{e}}_{2}+\sigma {\vec{e}}_{3}. \end{array}$$

The Frenet-Serret Equations of the curve $\tilde{\Gamma }$

(C.9)
$$\begin{array}{cc} \dot{\vec{\text{T}}} & =\vec{\text{N}},\\ \dot{\vec{\text{N}}} & =-\vec{\text{T}}+\sigma \vec{\text{B}},\\ \dot{\vec{\text{B}}} & =-\sigma \vec{\text{N}}, \end{array}$$

and

(C.10) $$\begin{array}{cc} \dot{\vec{\text{T}}} & =\vec{\Omega }\times \vec{\text{T}},\\ \dot{\vec{\text{N}}} & =\vec{\Omega }\times \vec{\text{N}},\\ \dot{\vec{\text{B}}} & =\vec{\Omega }\times \vec{\text{B}}, \end{array}$$

are (39) and (45). In this frame, $\vec{r}(\theta )$ is the original curve (function), and ${\vec{e}}_{3}(\theta )$ becomes its derived curve (function). In practice, it is difficult to find the physical meaning of the 3D curve $\vec{r}\left(\theta \right).$

Example C A circle on the unit sphere and its corresponding curve in the 3D space.

As shown in Figure 4(b), a circle on the unit sphere, marked as *C*, can be described as

(C.11)
$$\begin{array}{cc} & {\vec{e}}_{3}=\sin\;\vartheta_{0}\vec{\text{k}}+\cos\;\vartheta_{0}\cos\;\phi \vec{\text{i}}+\cos\;\vartheta_{0}\sin\;\phi \vec{\text{j}} \end{array}$$

with a fixed parameter *ϑ*_0_ ∈ (−*π*/2, +*π*/2) and a variable *ϕ* ∈ [0,2*π*].

Using the arc-length parameter *θ* ∈ [0,2*π*cos *ϑ*_0_], we have

(C.12)
$$\begin{array}{cc} & {\vec{e}}_{3}\left(\theta \right)=\sin\;\vartheta_{0}\vec{\text{k}}+\cos\;\vartheta_{0}\cos\;\frac{\theta }{\cos\;\vartheta_{0}}\vec{\text{i}}+\cos\;\vartheta_{0}\sin\;\frac{\theta }{\cos\;\vartheta_{0}}\vec{\text{j}}. \end{array}$$

Furthermore, we have

(C.13)
$$\begin{array}{cc} {\vec{e}}_{1}\left(\theta \right) & =-\sin\;\frac{\theta }{\cos\;\vartheta_{0}}\vec{\text{i}}+\cos\;\frac{\theta }{\cos\;\vartheta_{0}}\vec{\text{j}},\\ {\dot{\vec{e}}}_{1}\left(\theta \right) & =\frac{1}{\cos\;\vartheta_{0}}\cos\;\frac{\theta }{\cos\;\vartheta_{0}}\vec{\text{i}}-\frac{1}{\cos\;\vartheta_{0}}\sin\;\frac{\theta }{\cos\;\vartheta_{0}}\vec{\text{j}},\\ \sigma & ={\vec{e}}_{3}\cdot \left({\vec{e}}_{1}\times {\dot{\vec{e}}}_{1}\right)=\text{tg}\vartheta_{0},\\ \vec{\Omega } & ={\vec{e}}_{2}+\sigma {\vec{e}}_{3}=\frac{1}{\cos\;\vartheta_{0}}\vec{\text{k}}.\; \end{array}$$

Based on the definition (C.1), the corresponding 3D curve $\tilde{C}$ is

(C.14) $$\begin{array}{cc} \vec{r}\left(\theta \right) & =\theta \sin\;\vartheta_{0}\vec{\text{k}}+{\cos\;}^{2}\vartheta_{0}\sin\;\frac{\theta }{\cos\;\vartheta_{0}}\vec{\text{i}}\\ & \;-{\cos\;}^{2}\vartheta_{0}\cos\;\frac{\theta }{\cos\;\vartheta_{0}}\vec{\text{j}}, \end{array}$$

with the choice $\vec{r}\left(0\right)=-\vec{j}\;{\cos\;}^{2}\vartheta_{0}$.

Now, we see that $\tilde{C}$ is a helix, whose standard form is

(C.15)
$$\begin{array}{cc} & \vec{r}\left(\phi \right)=b\phi \vec{\text{k}}+a\;\cos\;\left(\phi -\frac{\pi }{2}\right)\vec{\text{i}}+a\;\sin\;\left(\phi -\frac{\pi }{2}\right)\vec{\text{j}}, \end{array}$$

with *a* = cos ^2^*ϑ*_0_,   *b* = sin *ϑ*_0_cos *ϑ*_0_,   *ϕ* ∈ [0,2*π*] (see Figure 4(c)). Its curvature and torsion are

(C.16)
$$\begin{array}{cc} & \kappa =1,\;\;\tau =\text{tg}\vartheta_{0}. \end{array}$$

The length of the helix $\tilde{C}$ for *ϕ* ∈ [0,2*π*] is $2\pi \sqrt{a^{2}+b^{2}}=2\pi \cos\;\vartheta_{0},$ which is equal to the circumference of circle *C* on the unit sphere. The radius of the helix $\tilde{C}(a={\cos\;}^{2}\vartheta_{0})$ is smaller than that of the circle *C*(cos *ϑ*_0_). The figure is not drawn to scale. The TNB frame rotates around the *z* direction at a constant velocity

(C.17)
$$\;\begin{array}{cc} & \vec{\Omega }=\frac{2\pi }{2\pi \cos\;\vartheta_{0}}\vec{\text{k}}=\frac{1}{\cos\;\vartheta_{0}}\vec{\text{k}}. \end{array}$$
